# Management of Traumatic Penile Degloving Injury in a Resource‐Constrained Setting: A Case Report and Literature Review

**DOI:** 10.1002/ccr3.73059

**Published:** 2026-07-03

**Authors:** Shehdev Meghwar, Umaya Memon, Vishan Das, Shankar Meghwar, Dhivya Bharathi, Siddhant Kumar Yadav

**Affiliations:** ^1^ Liaquat University of Medical and Health Sciences Jamshoro Pakistan; ^2^ Kazan State Medical University Kazan Russia; ^3^ Patan Academy of Health Sciences Patan Nepal

**Keywords:** degloving penile, penile degloving injury, resource‐limited area, surgical management

## Abstract

Genital degloving injuries from motorcycle accidents are uncommon, with most reported cases involving complete devascularisation requiring skin grafting. We present a 70‐year‐old male who presented to a rural health center following a high‐speed motorcycle accident with a unique distal pedicure circumferential penile skin avulsion. The penile skin sleeve remained viable through its distal attachment near the corona, while the proximal end was completely separated at the penoscrotal junction, exposing intact underlying corpora with confirmed urethral continuity. Management consisted of thorough wound irrigation, limited debridement, and simple anatomy reattachment of the viable avulsed skin using 4‐0 Prolene sutures, completely avoiding the need for skin grafts. The postoperative course was completed by a superficial bacterial infection on day 3, which resolved with oral linezolid and adjunctive serratiopeptidases. Follow‐up confirmed that vascular supply, normal sensation, unimpaired voiding, and preserved sexual function. This case demonstrates that in resource‐limited settings, meticulous assessment to confirm tissue viability enables conservative surgical management with excellent outcomes, avoiding the need for advanced techniques or grafts.

## Key Clinical Message

The case demonstrates that in resource‐limited settings, a favorable outcome in complex genitourinary trauma does not always require surgical techniques or skin grafts. This approach avoids the need for skin grafts or flaps, which may be unavailable or technically challenging in rural or low‐income centers.

**Abbreviations**
LMIC: low‐middle income country
PDI: penile degloving injury
RHC: rural health center
STSG: split‐thickness skin graft

## 1 Introduction

Penile degloving injury (PDI) is a rare traumatic condition in which the skin and superficial fascia of the penis are circumferentially avulsed, while the deeper structures, including the corpora cavernosa, corpus spongiosum, and Buck's fascia, are often preserved [1].

The injury usually results from shearing forces and is most commonly reported in young adult males, typically associated with occupational or industrial accidents, road traffic collisions, or sexual trauma. These injuries are also referred to as “power take‐off” injuries, reflecting their frequent association with rotating machinery where clothing or skin gets caught, leading to avulsion of the penile skin [2]. Overall, penile degloving injuries may range from partial to complete avulsion and, in severe cases, can be associated with urethral or adjacent genital structural injuries, underscoring the need for careful assessment [3]. The management of penoscrotal avulsion involves comprehensive cleansing and removal of nonviable tissues, followed by covering the exposed areas with healthy flaps from the surrounding skin. The embarrassment associated with the injury's location, cause, or circumstances frequently leads to delayed presentation, which can hinder both diagnosis and treatment [4].

We report the case of a 70‐year‐old man with a penile degloving injury in which, unlike typical degloving injuries that require skin grafting due to compromised skin viability, the penile skin retained an intact blood supply and was preserved without the need for graft reconstruction.

## 2 Case Presentation

### 2.1 Initial Case History/Examination

**FIGURE 1:**
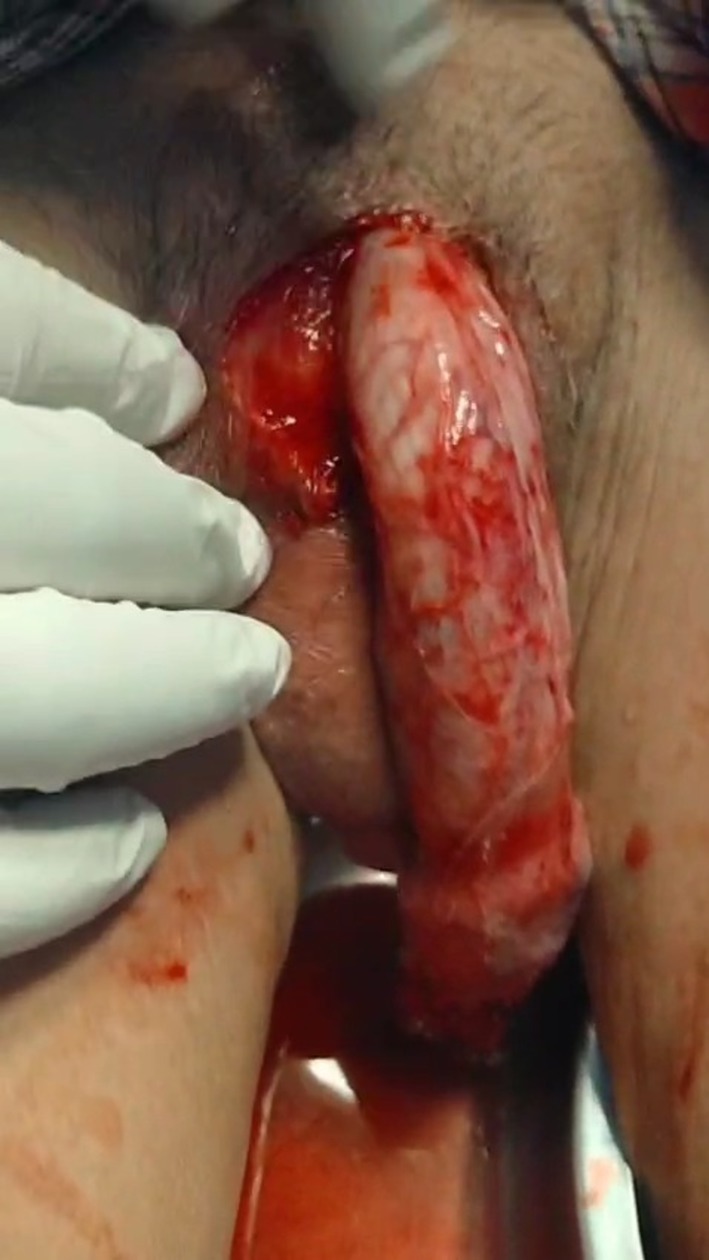
Penile degloving injury where the skin is separated from the base, with the distal portion still attached at the glans.

A 70‐year‐old male presented to the rural health center (RHC) following a high‐speed motorcycle accident. The patient reported losing control of the vehicle and striking the handlebars of the motorcycle with his perineum and genital region. He reported immediate severe pain and the sensation of his penile skin being pulled off. He denied loss of consciousness and inability to void at the scene. No other major traumatic injuries were reported. On examination, the patient was in mild distress but hemodynamically stable. Local genitourinary assessment revealed the circumferential avulsion of the penile skin sleeve, which remained pedicled and viable at its distal attachment near the corona of the glans penis. The avulsed skin was reflected proximally, hanging from this distal point, while the proximal attachment at the penoscrotal junction was fully separated (Figure 1). Consequently, the penile shaft was completely denuded, and the shaft skin was inverted, with its vascular supply intact via the preserved distal pedicle. The underlying corpora were exposed but intact, and urethral continuity was confirmed. The assessment of the extent of penile damage was done, and the viability of the testicles was confirmed. In the rural health center where resources are scarce, advanced imaging technologies were not readily available, so assessments relied primarily on clinical methods. The viability of the testicles was assessed through a physical examination that included checking color and integrity, palpating for consistency and tenderness, and confirming maintained blood flow without signs of ischemia or rupture. The continuity of the urethra was clinically assessed by ensuring that there was no blood at the urethral opening, confirming spontaneous urination, and ruling out urinary retention or signs of urethral injury. This unique presentation, which is a distally‐based, proximally degloved penile skin flap with preserved vascularity, deviates from the classic, completely devascularized penile skin.

### 2.2 Surgical and Postoperative Management

Management commenced with extensive wound cleansing using copious saline irrigation to achieve adequate decontamination, followed by careful debridement limited to nonviable debris, preserving the viable avulsed skin and optimizing the wound bed for definitive repair. The reconstructive approach was notably streamlined due to the favorable tissue conditions.

The preservation of viable penile skin and sparing of the scrotal tissue obviated the need for grafting.

**FIGURE 2:**
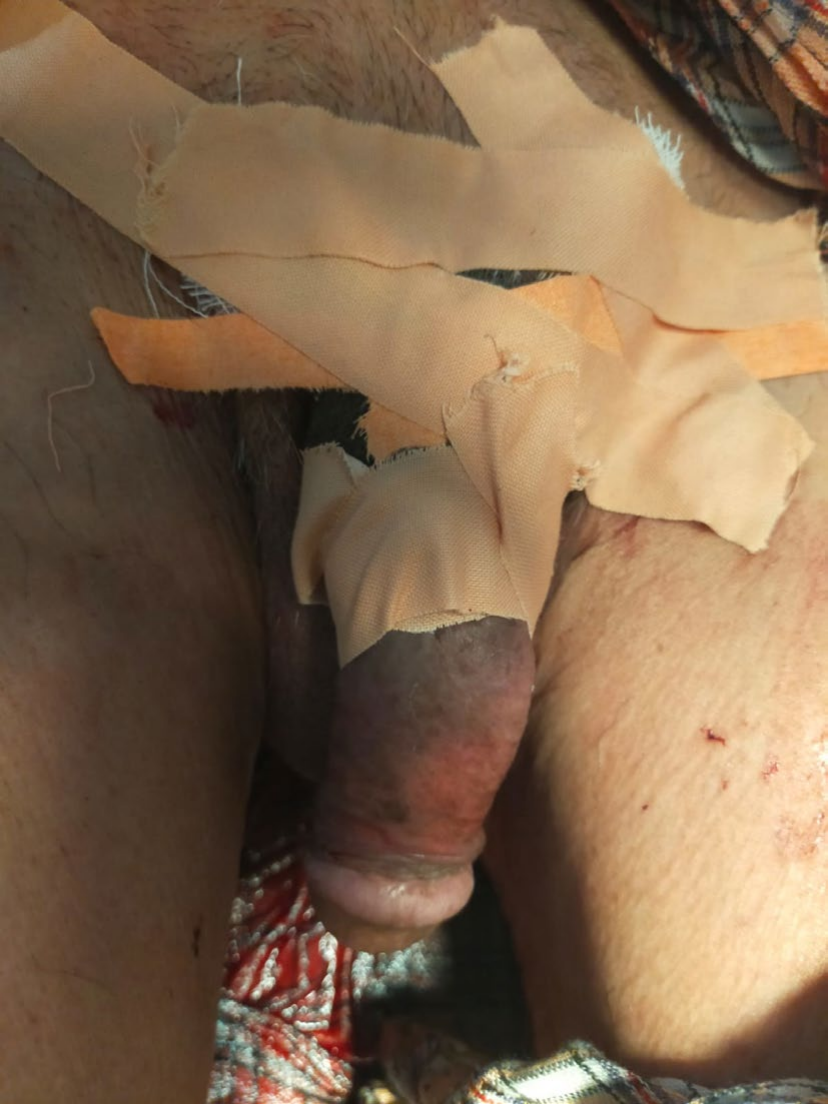
Penile degloving injury that was surgically treated and stabilized with surgical tape.

The avulsed penile skin was repositioned and securely reattached to the penile shaft using 4‐0 Prolene sutures (Figure 2). This anatomical preservation not only facilitated a more straightforward surgical reconstruction but also likely contributed positively to the patient's postoperative psychological outcome, particularly concerning future sexual function and aesthetic appearance.

**FIGURE 3:**
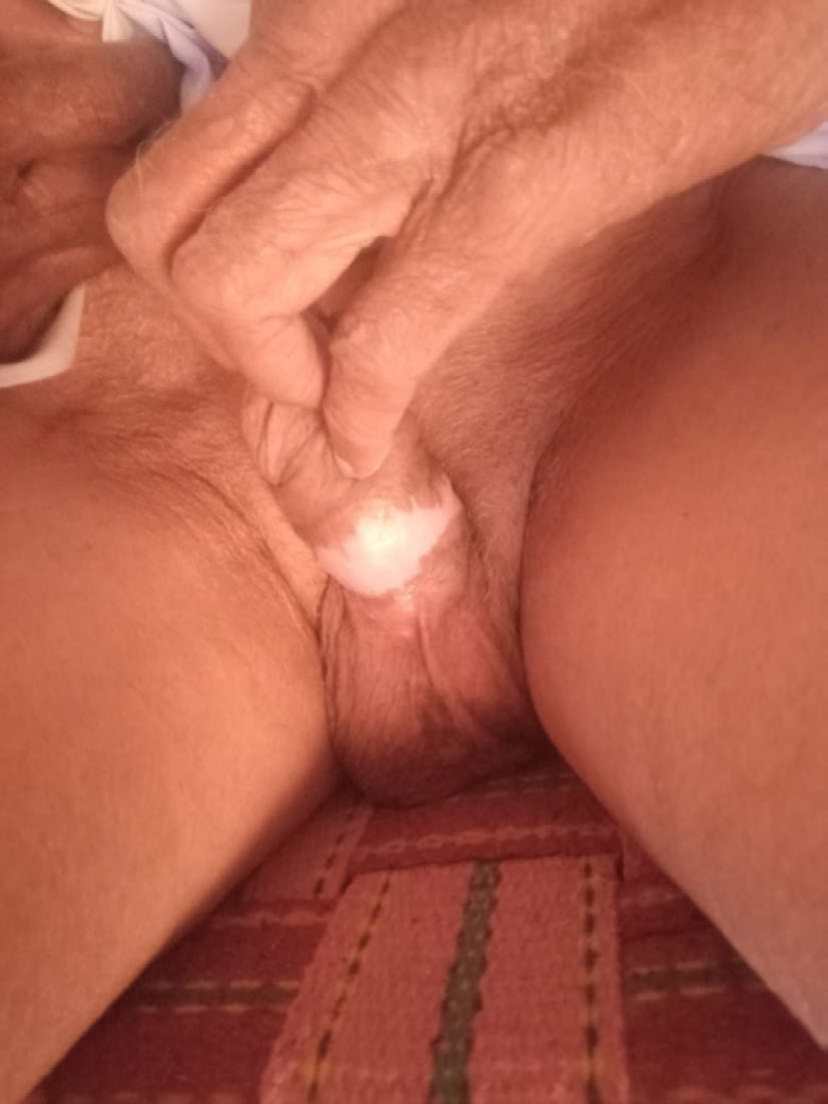
Complete recovery of the penile degloving injury at follow‐up, showing well‐healed tissue with satisfactory functional and cosmetic outcomes.

The patient remained hemodynamically stable during his hospital stay, with no signs of flap compromise, urinary problems, or wound separation. He was released after the wound condition improved, and proper urination was verified. The patient's recovery was uneventful concerning major surgical complications; however, a superficial bacterial infection developed at the surgical site on postoperative day 3. This was managed with a 1‐week course of oral linezolid, an oxazolidinone antibacterial agent, administered at 600 mg twice daily for its broad‐spectrum Gram‐positive coverage. Adjuvant therapy with the proteolytic anti‐inflammatory agent serratiopeptidase (10 mg, twice daily) was used to reduce swelling and facilitate tissue repair. The infection resolved fully with this intervention. Follow‐up assessments confirmed intact vascular supply, normal sensation, unimpaired voiding, and preserved sexual function throughout the postoperative period. Figure 3 illustrates the appearance of the penis during the follow‐up after complete recovery.

## 3 Discussion

This 70‐year‐old male presented to a rural health center with a unique, distally‐pedicled circumferential penile degloving injury from a motorcycle accident. Due to the preserved viability of the native skin, definitive reconstruction was achieved via primary closure, avoiding the need for grafting. The postoperative course was notable only for a promptly resolved superficial surgical site infection, and follow‐up confirmed excellent functional and aesthetic outcomes.

Traumatic injuries to the male external genitalia are comparatively uncommon relative to other anatomical regions. The predominant etiologies in such cases are occupational accidents, notably within the metalworking industry, and penetrating trauma from firearm discharge [5]. Although the present case is consistent with the literature regarding the mechanism of injury [4], namely a motor vehicle collision, its rarity is explained by two distinct aspects. First, the patient was of advanced age, whereas this injury pattern predominantly affects younger males [3, 6]; second, the degloved skin remained pedicled and viable, with perfusion maintained through its attachment to the distal aspect of the glans. This contrasts with the more common presentation of complete devitalization, which typically necessitates grafting as the primary reconstructive modality [2]. Given that our reconstruction did not require grafting, the patient was spared several potential postoperative complications commonly associated with graft‐based repairs. In situations where grafting is unavoidable, the primary surgical challenge becomes preventing graft contracture, a complication that can significantly affect both function and cosmesis. As described by Li et al. [7], a similar injury necessitated a graft, and the team employed a combination of strategies to mitigate this risk, including supplementation with local tissue flaps, intentional penile immobilization, and vacuum‐assisted closure. This approach aimed to maintain anatomical shape, enhance graft survival, and reduce contracture, thereby preserving both sexual function and aesthetic appearance. In contrast, the favorable anatomy in our case, with viable native skin precluding the need for a graft, allowed for a more straightforward primary closure. This likely contributed to the patient's uncomplicated recovery and favorable psychological outcomes related to body image and sexual function, without requiring the complex adjunctive measures. Managed in a low‐resource LMIC setting, this case benefited from a reconstructive approach that inherently favored resource constraints. The viability of the degloved skin permitted primary closure, circumventing the need for grafts and their attendant complexities. This not only simplified postoperative care but also reduced costs and length of stay, key considerations in environments with limited surgical infrastructure. Table 1 summarizes clinical characteristics, mechanism of injury, management approaches, and outcomes of PDI cases reported from different regions.

**TABLE 1:** Literature review of previously reported cases of penile degloving injuries.

Study	Sex—age	Cause	Injury type	Was graft needed (if yes, then which site was used)	Was there any urethral injury	Postoperative infections
Bhattarai et al. (2020) [4]	M—28 years	Motor vehicle accident	Penoscrotal degloving injury	Yes, full‐thickness skin graft (donor site not specified)	No	N/R
Mathur et al. (2010) [8]	M—8 years	Dog bite	Penile skin	No	No	Not occurred
Alkahtani et al. (2020) [2]	M—26 years	Tractor entanglement	Penoscrotal degloving	Yes, split‐thickness skin grafts (STSG) from right thigh	No	N/R
Górka et al. (2025) [6]	M—8 years	Unwitnessed fall from farm equipment	Degloving Penoscrotal	No	No	Not occurred
Thompson et al. (2019) [3]	M—14 years	Blunt trauma from a bicycle collision	Groin, scrotum and penis	No	No	N/R
Bhogesha (2019) [9]	M—54 years	Machine entanglement	Isolated penile	Yes, thick splits kin graft from Not specified site	No	Not occurred
Zanettini et al. (2005) [10]	M—30 years	Industrial machine accident	Penoscrotal	Yes, split‐thickness skin graft from anterosuperior area of the iliac spine	N/R	N/R
Jena et al. (2007) [11]	M—28 years	Road traffic accident	Traumatic degloving injury of penile and scrotal skin	No	No	N/R
Li et al. (2018) [7]	M—50 years	Clothes entangled in a grinding machine	Penile and scrotum skin	Yes, graft from left medial thigh	No	Not occurred
Paraskevas et al. (2003) [12]	M—40 years	Agricultural machine	Penoscrotal	No	yes	NR
Selvan et al. (2009) [13]	M—27 years	Agricultural machine accident	Penis and scrotum	Yes, right thigh	No	N/R
Shetty et al. (2008) [14]	M—24 years	Run over by heavy vehicle tyre	Penis and scrotum	No	N/R	The wound swab after 4 days grew *Staphylococcus aureus* , later died

## 4 Patient's Perspective

The patient felt considerable distress and fear right after the injury because of the intense pain and the unusual look of the injured area. Coming from a resource‐limited environment, he worried about whether suitable medical care would be available. After receiving immediate assessment and surgical intervention, the patient felt a sense of relief and comfort due to the thorough explanation of his condition and treatment strategy. As he continued to recover and the wound fully healed, his anxiety slowly diminished.

## 5 Take‐Home Message

Penile degloving injuries are uncommon yet significant urological and genital emergencies that necessitate prompt identification and surgical intervention to maintain functionality and achieve desirable cosmetic results. Quick treatment, proper wound management, and suitable reconstructive methods are essential for the best recovery. This case underscores the necessity of timely referrals and specialized care, especially for individuals coming from resource‐constrained environments, to avoid complications and guarantee acceptable functional and psychological results.

## 6 Conclusion

This case illustrates that meticulous initial assessment to confirm tissue viability is the cornerstone of managing complex genitourinary trauma and resource‐limited settings. When vascularity is preserved, conservative surgical management achieves excellent outcomes without advanced techniques, reducing costs and hospitalization. It reinforces that technology is not always the answer; clinical judgment and tissue preservation are.

## Author Contributions

**Shehdev Meghwar:** conceptualization, data curation, supervision, project administration, writing – review and editing, writing – original draft, validation. **Umaya Memon:** project administration, validation, visualization, writing – review and editing, writing – original draft. **Vishan Das:** validation, visualization, project administration, supervision, writing – original draft, writing – review and editing. **Shankar Meghwar:** validation, project administration, visualization, writing – review and editing, writing – original draft. **Dhivya Bharathi:** visualization, validation, writing – review and editing, writing – original draft. **Siddhant Kumar Yadav:** visualization, validation, writing – original draft.

## Funding

The authors have nothing to report.

## Ethics Statement

The authors have nothing to report.

## Consent

A written informed consent was obtained from the patient based on the journal's policies.

## Conflicts of Interest

The authors declare no conflicts of interest.

## Data Availability

Data sharing is not applicable to this article as no datasets were generated or analyzed during this study.
